# COVID-19 and food security in Sub-Saharan Africa: implications of lockdown during agricultural planting seasons

**DOI:** 10.1038/s41538-020-00073-0

**Published:** 2020-09-14

**Authors:** Ayansina Ayanlade, Maren Radeny

**Affiliations:** 1 Department of Geography, Obafemi Awolowo University, Ile-Ife, Nigeria; 2 CGIAR Research Program on Climate Change, Agriculture and Food Security (CCAFS), International Livestock Research Institute (ILRI), Nairobi, Kenya

**Keywords:** Environmental impact, Agriculture

## Abstract

COVID-19 pandemic movement restrictions as part of the control measures put in place by countries in Sub-Saharan Africa (SSA) has implications on food security, as movement restrictions coincided with planting periods for most of the staple crops. The measures are affecting important staple crops in SSA, and are likely to exacerbate food security challenges in many countries. Achieving adequate food supply in SSA requires developing better policies and packages to confronting the challenge of reducing hunger post COVID-19 pandemic. The lessons learned after COVID-19 crisis will be very important for African countries to rethink their strategies and policies for sustainable economic growth, as COVID-19 many have significant impacts on all sectors of their economies.

## Introduction

Sub-Saharan Africa (SSA) is one of the most vulnerable regions to the social and economic impacts of COVID-19. Vulnerability of SSA is attributed to several factors including; poor health facilities in many SSA countries and low capacity for testing, timely detection and response to COVID-19 cases^1,2^. In particular, the initial movement restrictions (complete and partial lockdown) imposed by countries coincided with the planting periods (important in the agricultural calendar) for most of the staple crops in the region. SSA accounts for nearly 13% of the population globally, with the proportion of the population living in poverty and undernourished remains high among the rural communities^3^. Agriculture remains the main source of livelihood^4,5^ and food security for majority of the rural population in SSA, with the climatic conditions favouring cultivation of diverse crops. Agricultural production is mainly rain-fed, with pockets of irrigated land. For example, Western Africa accounts for more than 60% agricultural output from the SSA, over the past 24 years, but Land degradation and climate change are now posing additional threats to agriculture^6–8^. In terms of agricultural sector spending, less than 20% of the SSA countries have achieved their commitments as per the Malabo Declaration on accelerated agricultural growth and transformation and the Comprehensive Africa Agriculture Development Programme (CAADP) and the situation is likely to worsen with the COVID-19 movement restrictions put in place by African countries. The movement restriction measures adds to the hardships and challenges faced by nearly 1.3 billion people in Africa^2^, especially those working in the informal sector, the larger percentage of these people rely on daily wages and are living in poverty, with less than one dollar a day^9^.

In particular, the months of March and April are the planting periods for some of the important staple crops in SSA and very significant in the cropping calendar, though there is temporal and spatial variability in planting time^10–12^. Many of the crops in SSA are cultivated under rain-fed conditions, thus the timing of the planting is very important and delay in planting during these months as may have been experienced as a result of the movement restrictions may significantly affect crop growth and lead to food shortage for the year. While planting periods vary by agro-ecological zones and determined by climate, they have the potential to affect agricultural production in SSA^13^. Currently, the potential impacts of COVID-19 crisis on agriculture in SSA are unclear, including potential impacts on the agricultural value chain. What is obvious at present is that COVID-19 is disrupting activities of farming communities, with potential negative impacts on agricultural production. Over 65% of the households in SSA are mainly smallholder farmers, many are poor and vulnerable. Thus, many African governments have developed some measures to help vulnerable households during the COVID-19 lockdown. Such measures include distribution of grains to vulnerable households, especially the poor households. The measures among others are aimed at meeting the urgent immediate food needs of the population. However, many farmers do not have access to critical inputs during the lockdown; this will have a negative impact on 2020 agricultural production. Consequently, the COVID-19 crisis may have potential negative impacts on food security in SSA countries.

**Fig. 1:**
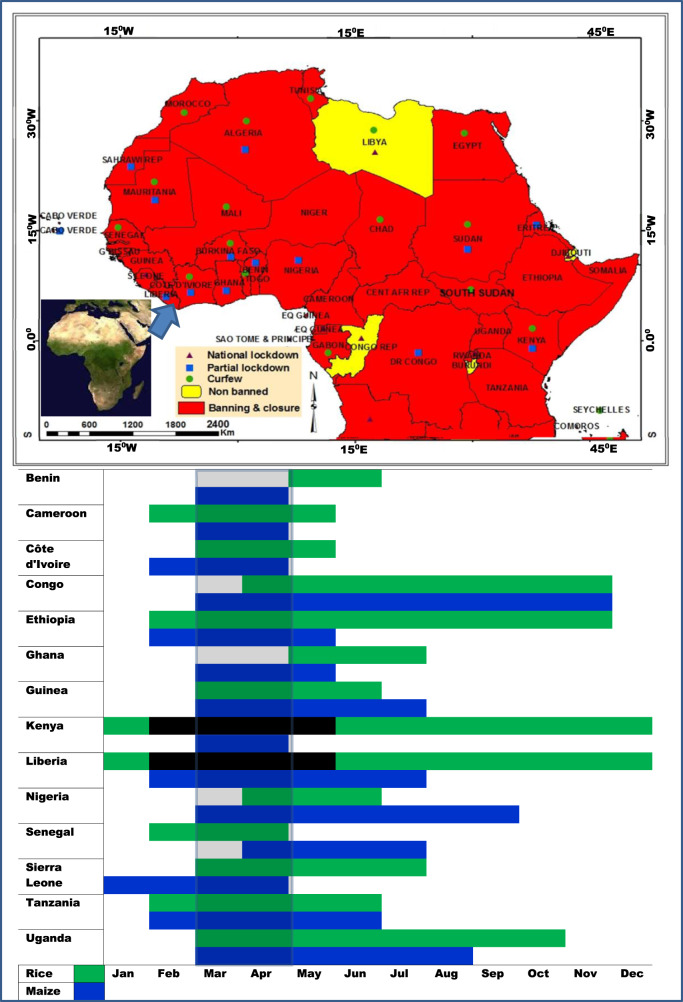
The potential crops planting impacts of COVID-19 movement restrictions. Movement restrictions during the planting periods may increase the potential for food shortage. Nearly 65% of farmers in the countries are smallholder farmers who start rice planting (in green) and maize (in blue) planting mostly in March and April, a period overlapped with COVID-19 movement restrictions.

This paper examines the potential impacts of COVID-19 movement restrictions on food security in SSA, focusing on the effects on planting days (season) for major staple crops, including effects through restricted access to important farm inputs. We examine the potential impacts of movement restrictions on planting dates of rice and maize in major producing countries in SSA (Fig. 1). Rice and maize are widely cultivated crops for food and income, as they have a high market potential in SSA countries. Both rice and maize are now fastest-growing staples food crops in Africa^14^, though there are problems of pathogens and pests on major food crops in the region^15^. Among the cereal crops produced, maize and rice have severe implications for economic development in most SSA countries, as their contribution to agricultural GDP is high. Over the past decades, these crops have become important in the diets of many people in SSA. In recent years, African governments have developed initiatives to enhance rice cultivation and increase the production capacity of local farmers, especially in smallholder systems.

## Implications of COVID-19 movement restrictions on rice and maize production

As indicated in previous section, agricultural production systems in many SSA countries are mainly rain-fed. Following the timely onset of seasonal rains, March to mid-April is the preferred planting period for many rice and maize farmers in SSA (Fig. 1). The data for planting period used in this study were sourced from the crop statistics recorded by FAO–FAOSTAT, while COVID-19 data were collected from the Africa CDC (2020)^16^. There are regional differences in the relative crops’ planting date and this reflects agro-ecological, climatic and cultural diversity. What is the hope for ample yield of rice hereafter COVID-19 lockdown? Production and supply of rice and maize in many SSA countries are expected to substantially decline due to projected global recession^17^ and higher transaction costs, as combined effects of COVID-19 lockdown. Maize and rice are important in the continent as the major staple crops for most SSA countries^18^. While crop production practices in Africa are diverse, what is clear is that in most cases the local production does not adequately meet the demand of the ever-increasing population. In recent years, Nigeria is the largest rice-producing country in Africa. The major drivers for the increase in rice production in recent years, in some SSA countries, are very clear. In Nigeria, for example, expansion of rice production is as a result of government efforts in enhancing agrarian production in the country, with nearly 85% of the Nigerian States engaging in rice cultivation. In Eastern Africa, increasing crop production and productivity is a top priority for economic development in Kenya and Tanzania. These countries are the main rice producers in East Africa. Despite the increase in rice crop production in recent years, many SSA countries still import rice and the countries majorly earn their total foreign exchange revenue from agriculture. The majority of the rice produced in SSA countries are consumed locally but the consumption rises by about 6% annually^19–23^.

Despite being major staple crop in Africa, studies have shown that the local rice production and yields are very low compared to other parts of the world, partly attributed to the fact that rice is mainly grown by smallholder farmers in many African countries^19,20^. For many African countries, the planting periods for rice and maize is usually in March to mid-April, a period overlapped with COVID-19 movement restrictions (Fig. 1). Reports indicate that rice importation fluctuated substantially in recent years, still the demand for rice consumption in Africa has surpassed the local production capacities and the COVID-19 crisis, however, may add to this scenario in 2020^24^. Overall, the lessons learned post COVID-19 crisis will be very important for African countries to rethink their strategies and policies for sustainable economic growth, as COVID-19 may have significant impacts on all sectors of the economies.

## Border closures, labour availability during COVID-19 movement restrictions and the implications for the food system

**Fig. 2:**
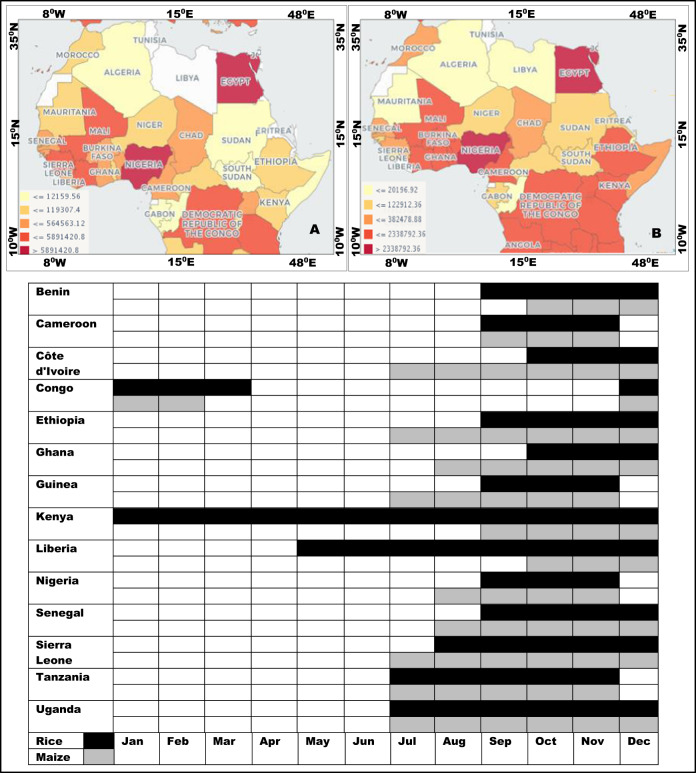
The production quantities maps and information on harvesting periods of rice and maize in SSA countries between 1994 and 2018. Nearly 65% of farmers in the SSA countries are smallholder farmers who harvest rice (**a**) and maize (**b**) mostly in July and December. Harvesting periods is likely to be affected by COVID-19 movement restrictions.

The movement restrictions put in to reduce the spread of COVID-19 period affect labour mobility and availability, especially for the predominantly labour-intensive agricultural production in SSA. The agricultural sector employs 70% of the total workforce in the region, making it the most important sector for livelihoods and economic development^25^. The planting period is the period with the peak labour demand in the agricultural calendar^26^. Low labour supply or labour shortage, has significant implications on food security and the economy is likely to be severely negatively impacted^25^. Most of the countries in SSA implemented some form of border closure during the planting month of maize and rice, and this also prolonged the start of the harvest period (Figs. 1 and 2). The data for the crop harvesting periods, used in this study, were sourced from the crop statistics recorded by FAO. The crop statistics records of production quantities are available in http://www.fao.org/agriculture/seed/cropcalendar/welcome.do; while the data on harvesting periods were collected also from statistics records of FAO which are available in http://www.fao.org/faostat/en/#data/QC/visualize. There is a high probability that the crop harvests will be compromised due to the restriction in movements during the COVID-19 and low labour force. In addition, these measures also affected the supply or access to essential farm inputs such as fertilizers and pesticides. As labour shortages are imminent in the SSA, many people that derive their daily wages from farming and non-farm informal sector are therefore, likely to lose their jobs and income, with long-term implications and effects on the economy of the region.

While border closures is an important measure to protect citizens, according to WHO almost all the countries in the SSA are major importers of food. Some limited flights were allowed to facilitate the supply of protective and life-saving equipment to affected countries, allowing humanitarian cargo or emergency flights. In most cases, the border closures have led to the shortage of essential food supplies, while the price of the available food significantly increased. In addition, the border closures limit the supply and increase the price of related goods and services, including essential farm inputs such as supply seeds and agrochemicals, pesticides, and fertilizers majority of which are imported by SSA countries^25,27^.

The long- term implications of border closures for key food commodities such as rice and maize cannot be overstated. The larger proportion of these food crops are still imported in many of SSA, with border closure likely to aggravate Africa’s fragile economic situation^28^. The majority of the countries in SSA are particularly vulnerable to the COVID-19 pandemic because of over-reliance on food imports (such as rice and maize), with high poverty rates. Besides, border closures are likely to have adverse effects on the supply of seeds and agrochemicals, pesticides, and fertilizer as the majority of them are imported to SSA countries^25^.

## Environmental and pest challenges overlapped the movement restrictions

The effects of the COVID-19 pandemic is aggravated not only by border closure, which leads to low importation of food and raw materials, but also by the other environmental problems in the SSA. The most significant environmental challenges for maize and rice production include climate change and variability, pests, and other emerging crop diseases. These environmental challenges have impacts on food security for the region. For the agriculture sector, farmers have experienced change in climate in recent times. The adaptation measures to climate changes include; adjusting farm management practices as well as changing cropping calendars to optimize the use of available water for crop growth, but such changes do have impacts on crop yield for the year^29^. Hitherto, climate change has become a key challenge to the economies of different countries in SSA. Rain-fed agriculture forms the major activity of livelihood for about 70% of the population^30^. The variation in climate does not provide a conducive environment for farming as rainfall is the main source of water for the production of crops. With many economies depending on agriculture, most especially the SSA countries, the change affecting agriculture invariably affects the economy of the SSA countries.

Studies have reported that climate change and variability manifested in changing rainfall patterns and temperature, determines water availability for growth and production of crops with direct effects on crop yields^31–34^. An increase in global temperature by 2 °C, for example, can potentially lead to a 17% reduction in crop yields^35,36^, most especially those very sensitive crops, such as maize and rice, and this may result in reduction of yields in SSA^37,38^. In the case of maize for example, though the seed germinates at a temperature range of 18–21 °C, it can be grown in both rainy and dry seasons provided adequate water is available during germination and the first month of growth. Thus, nearly 85% of the maize acreage is under rain-fed conditions during the monsoon when over 80% of the annual rainfall is received^39^. Depending on the climate and water availability, rice can be grown in all seasons (i.e., rainy and dry seasons). In addition, depending on the rice variety crop, duration of maturity varies from 100 to 150 days. Rice cultivation is possible with a good rainfall ranging between 100 and 200 mm and about 125 cm during the growing season but there must be less or no water at ripening stage. The temperature also should be fairly high ranging from 20 to 40 °C with maximum day time temperature not exceeding 30 °C and minimum 20 °C^40^. Since COVID-19 has occasioned an emergency, rain-fed agriculture remains the main source of food production in Africa and farming under uncertain climate variability has remained a challenge to the farmers as well discouragement to those who would have invested in farming because of the negative impacts of climate change^41–43^. Various strategies are used by farming communities in SSA to cope with climate change and variability and these include harvesting of rainwater for irrigation, soil and land conservation measures, intercropping, growing earlier maturing crops and crop varieties, crop diversification, migration of farmers to more productive areas among others. While SSA countries have very high potential for maize and rice production (Fig. 2), the impacts of climate change^44–46^ and recent locust invasion^47,48^ coupled with COVID-19 movement restrictions are likely to reduce the production potential^49^.

In addition to the environmental challenges highlighted above, the recent invasion of the desert locust, which were partly attributed to climate change, overlapped with the COVID-19 movement restriction period. Many SSA countries have been experiencing food insecurity resulting from drought and locust invasion, especially in the Horn of Africa, with effects on economic growth. The COVID-19 movement restrictions may have longer-term implications. The situation for many smallholder farmers may perhaps even be more pressing and urgent, with the failure of this year’s harvest (Fig. 2), leading to a financial hardship that could significantly impact on the success of subsequent seasons, such as, insufficient capital for buying seeds, fertilizers, pesticides, and general land management. COVID-19 movement restrictions limits social interactions and closure of certain sectors of the economy where farmers could get financial aid. Since the majority of SSA countries are dependent on agriculture, there is a high probability that the region may be threatened by the first serious recession in the region in 25 years, as the economic growth in the region will decline from 2.4% in 2019 to −2.1 to −5.1% in 2020^50^. It has been reported that many households in SSA, especially in rural communities, may be directly impacted by the predicted decline in economic growth, as the supply-side economic shocks such as those caused by the COVID-19 pandemic^51^.

## Conclusions

The is a need for governments at all level in SSA to develop better policies and strategies for reducing hunger post COVID-19 pandemic in Africa and improving food security^52–54^. All counties in SSA need to act together immediately, under African Union to respond and prepare a recovery plan post COVID-19 pandemic, to improve food supply in the continent, as the UN system, through UNDP, will provide support to the countries through international response^55–57^. If appropriate actions are not taken, it is projected that some farmers may switch crop types, while some young farmers may move out of agriculture completely^5^. Farmers in SSA regions may need to adjust the seasonal calendar to be suitable to these changes, and organize planting calendar based on information from warning system and traditional knowledge in production is crucial to maximizing optimal conditions^58^. This proposed option may not totally guaranty maximum yields of maize and rice this year (2020), as seems to be unsuitable for the current climate change condition. Thus, the integrated approach to the meteorological science and crop science, with traditional/indigenous knowledge in the early warning system, is the best way to determine the appropriate seasonal calendar. Therefore, short-term seasonal weather forecasting is one of the options for adjusting the seasonal agricultural calendar suitable for the annual change^59–61^.

Even though COVID-19 is an unprecedented crisis, African leaders and governments are urged to use COVID-19 pandemic crisis as an opportunity to offshoot change to shaping the development of the agricultural sector for food security nevertheless the pandemic is much more health issue. The governments have recognized the challenge of potential food shortage and responded aggressively to meet the food need of their population. This will aid to keep the food value chain alive if governments in SSA countries focus on key logistical bottlenecks. The majority of smallholder farmers in SSA need cash hand-outs and safety net programmes, while the bank may need to extend payment deadlines and wave interest on farmers’ loans and rural households loans. There is no doubt that food security and safety net policies and programmes need to be enhanced in Sub-Saharan Africa.
